# Hinman Syndrome: A Rare Entity With Neurogenic Bladder-Like Symptoms

**DOI:** 10.7759/cureus.58191

**Published:** 2024-04-13

**Authors:** Sravani Gampala, Leen Alkukhun, Zohaib Khan, Ravikumar Hanumaiah, Anand Majmudar

**Affiliations:** 1 Radiology, State University of New York (SUNY) Upstate Medical University, Syracuse, USA

**Keywords:** intermittent encoperesis, pinecone bladder, christmass tree bladder, neurogenic bladder, hinnman syndrome

## Abstract

Hinman syndrome, as is the case with many other rare conditions, is a disease very commonly under-considered or missed in the diagnosis of patients with the presenting symptoms. Clinical and radiographic manifestations of the condition are easily confused with neurogenic bladder without proper history collection and neurological examination. Patients typically present with symptoms including enuresis, urinary retention, reoccurring urinary tract infections, and encopresis. Imaging often shows hydroureteronephrosis and marked bladder wall thickening. While these signs are characteristic of neurogenic bladder, routine neurologic examinations and MRIs of patients with Hinman syndrome often show normal results, and their condition is currently thought to be an acquired behavioral and psychological disorder, often associated with abnormal family dynamics. We present the case of a 14-year-old boy, who presented to the emergency department with nausea, bilateral flank pain, and urinary retention. The patient had an over seven-year history of recurrent urinary tract infections (UTI) and intermittent encopresis and followed up with different providers. Due to the patient’s extensive history and the failure of previous treatments, he was evaluated for causes of neurogenic bladder, but the MRI of the lumbar spine was normal. Fluoroscopic voiding cystourethrogram (VCUG) was ordered and demonstrated abnormal and trabeculated contour of the urinary bladder with bilateral vesicoureteral reflux consistent with the diagnosis of Hinman syndrome.

## Introduction

Nonneurogenic neurogenic bladder syndrome, now referred to as Hinman syndrome, is a rare condition that typically presents in early to late childhood. Hinman syndrome is characterized by urinary bladder dysfunction and clinical and radiologic features associated with a neurogenic bladder but with no signs of neurologic damage [1]. Children’s symptoms include dysfunctional voiding involving detrusor overactivity and inability to relax the external urethral sphincter, with many cases progressing to encopresis and urinary tract infections. Radiologic signs usually exhibited are a bladder with a "Christmas tree" or pine cone appearance, bladder wall thickening and trabeculation, unilateral/bilateral hydroureteronephrosis, and vesicoureteral reflux [2]. Identification and diagnosis of Hinman syndrome in children is crucial to avoid further complications, including urinary tract infection (UTI) and renal damage. Recommended treatment for this condition is still up for debate, but recommendations consist of suggestion therapy including hypnosis and bladder retraining, biofeedback, anticholinergic drugs, and surgical procedures in severe cases [1,3].

## Case presentation

A 14-year-old boy was referred from an external hospital due to nausea bilateral flank pain, and urinary retention. His medical history was notable for recurrent urinary tract infections since the age of six, constipation treated with MiraLAX, and voiding dysfunction managed with biofeedback and pelvic floor muscle training exercises. Notably, a voiding cystourethrogram at the age of seven revealed left-sided grade 3 vesicoureteral reflux, right-sided grade 1 vesicoureteral reflux, and a potentially narrowed segment in the membranous urethra. By the age of 13, a renal ultrasound demonstrated severe bilateral hydroureteronephrosis and irregular bladder contour.

On a current visit, he presented with bilateral flank pain and urinary retention. His labs showed acute kidney injury with a creatinine of 3.5 mg/dl and blood urea nitrogen (BUN) of 29 mg/dl (baseline creatinine 0.6mg/dl and BUN 18 mg/dl). The patient was already on oxybutynin, tamsulosin, and nitrofurantoin for urinary tract infection prophylaxis. Further investigations of CT abdomen and pelvis showed bilateral hydroureteronephrosis and a thickened urinary bladder wall. In addition, an MRI lumbar spine performed to evaluate for neurologic deficits showed normal findings. Meanwhile, the psychological evaluation of the patient was positive for anxiety, depression, obsessive-compulsive disorder, and attention-deficit hyperactivity syndrome; he was being treated with sertraline, dextroamphetamine/amphetamine and was following up with a psychotherapist and psychiatrist on an outpatient basis.

After years of poor follow-up and given that his history of urinary and fecal retention was mainly attributed to behavioral etiology, the patient was diagnosed with Hinman syndrome.

**Figure 1:**
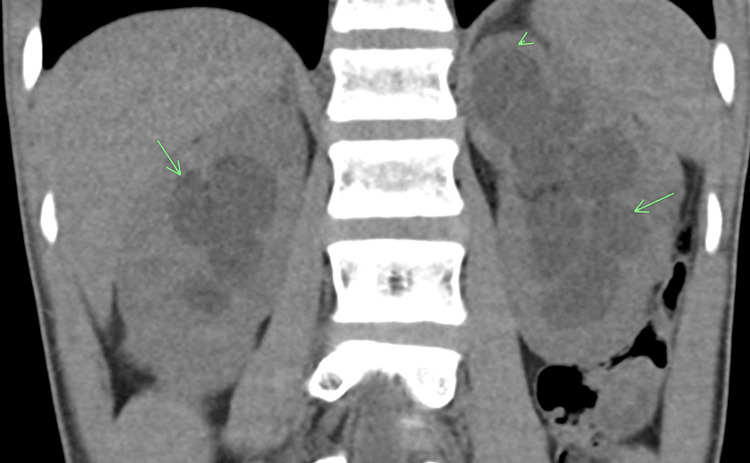
Computer tomography (CT) of the abdomen and pelvis Coronal non-contrast CT image of the abdomen showed bilateral hydroureteronephrosis (green arrows) with left upper pole renal cortical thinning (arrowhead).

**Figure 2:**
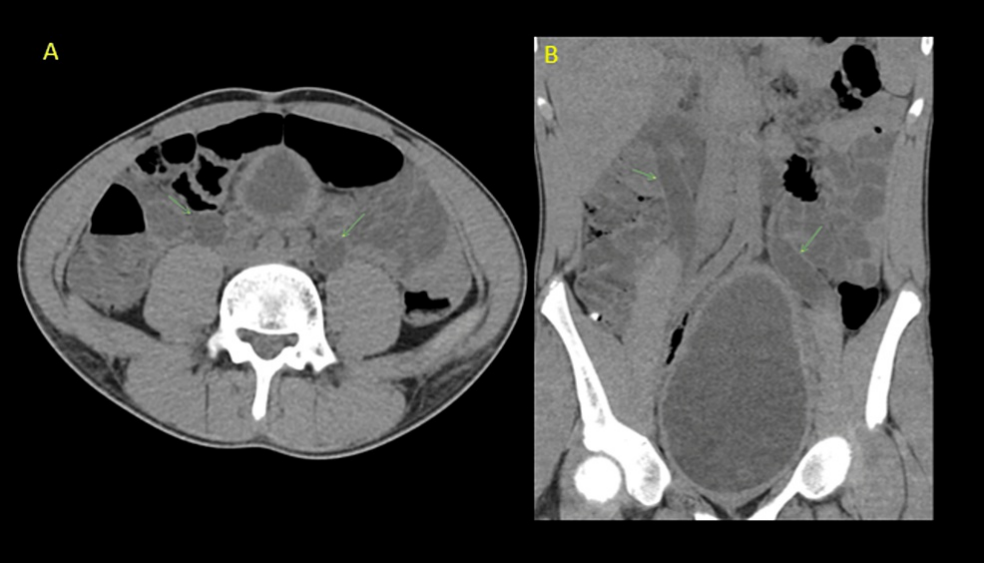
Computed tomography (CT) of the abdomen and pelvis Axial (A) and coronal (B) non-contrast CT images of the abdomen demonstrated bilateral hydroureteronephrosis (arrows).

**Figure 3:**
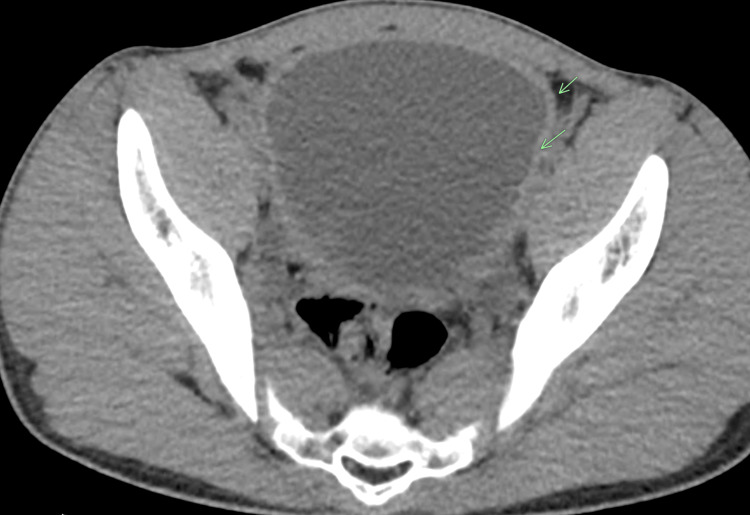
Computed tomography (CT) of the abdomen and pelvis Axial non-contrast CT image of the pelvis demonstrated urinary bladder wall thickening.

The following are the imaging features of various radiological modalities in this patient. Computed tomography (CT) of the abdomen and pelvis performed at that time showed bilateral hydronephrosis and a thickened urinary bladder wall (Figures 1-3).

**Figure 4:**
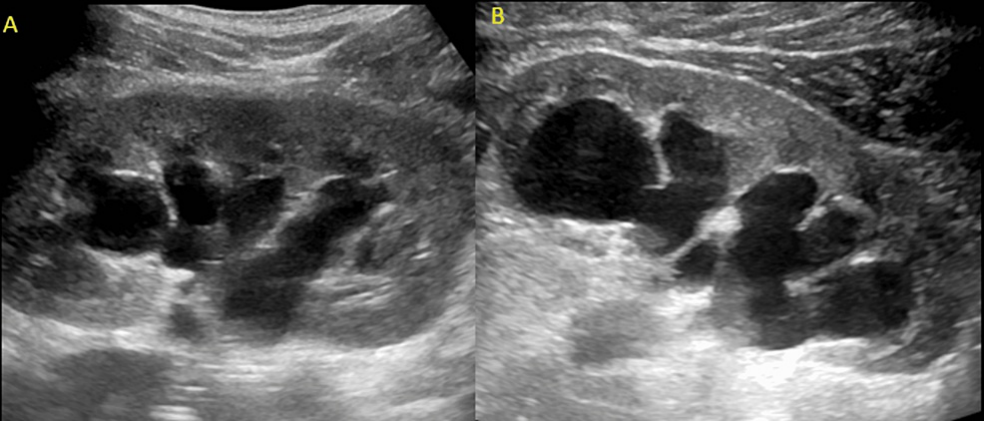
Grayscale ultrasound images of the kidneys Sonographic evaluation of the right (A) and left (B) kidneys showed bilateral hydronephrosis.

**Figure 5:**
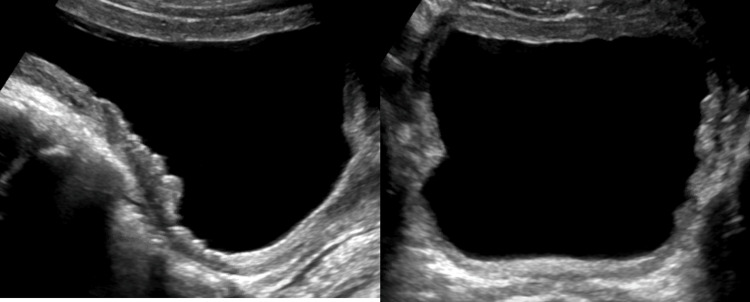
Grayscale ultrasound images of the urinary bladder. Sonographic evaluation of the Urinary bladder demonstrates a thickened and trabeculated urinary bladder wall.

Renal ultrasound at that time showed bilateral hydronephrosis, trabeculations, irregular contour of the urinary bladder, and thickened bladder wall (Figures 4-5).

**Figure 6:**
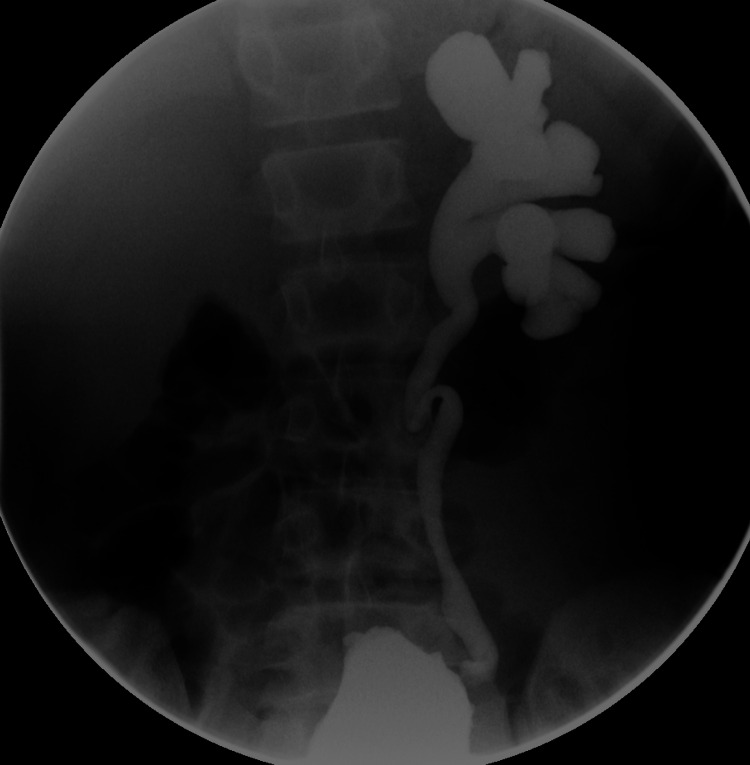
Fluoroscopic voiding cystourethrogram Fluoroscopic voiding cystourethrogram demonstrated left vesicoureteral reflux.

**Figure 7:**
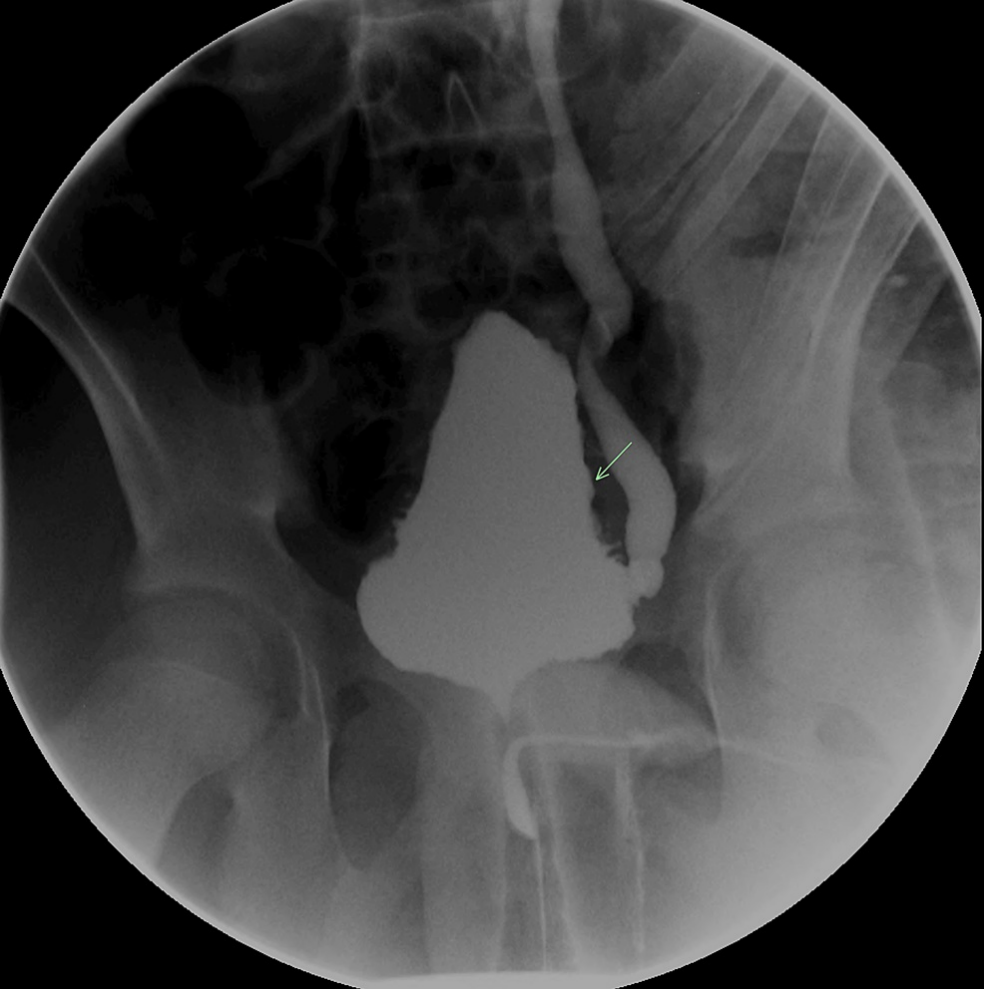
Fluoroscopic voiding cystourethrogram Fluoroscopic voiding cystourethrogram demonstrated a trabeculated urinary bladder wall.

Fluoroscopic VCUG showed progression of the vesicoureteral reflux to grade 4 and a trabeculated urinary bladder contour (Figures 6-7).

**Figure 8:**
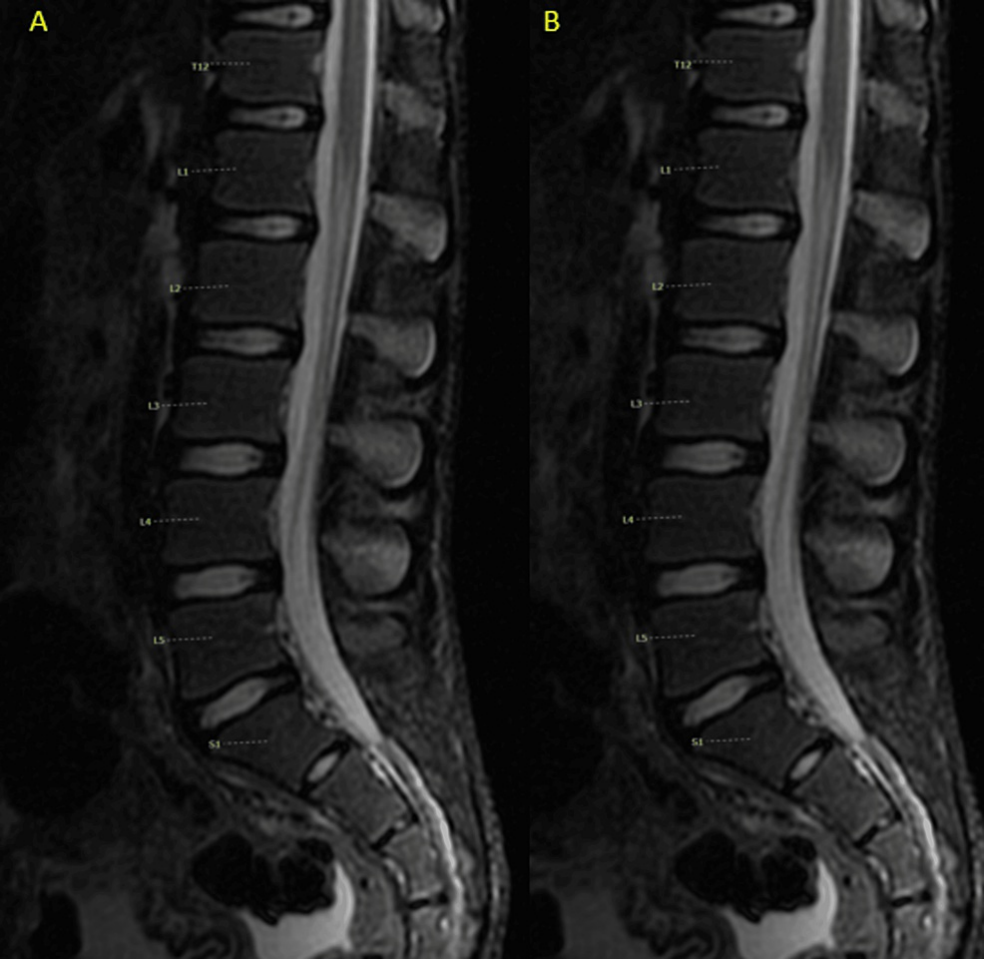
MRI of the lumbar spine T2 (A) and T2 STIR (B) demonstrated a normal-appearing cord and filum, no abnormal signals, and no evidence of disc bulges or spinal canal stenosis.

Lumbar spine MRI evaluation for the neurologic deficit was normal (Figure 8).

## Discussion

Hinman syndrome was initially described by Hinman in 1971 in an eight-year-old male who presented with day and night wetting, urinary tract infection, and radiologic evidence of urinary bladder and upper urinary tract abnormalities triggered solely by psychological malfunction. In 1973, Hinman and Baumann described non-neurogenic neurogenic bladder after the study of 14 boys between the ages of 8 and 11 years old who presented with recurrent urinary tract infections, urinary incontinence, enuresis, encopresis, and upper urinary tract dilatation without any evidence of neurologic dysfunction and these abnormalities were all related to behavioral abnormalities [2]. The syndrome was then renamed after Hinman in 1986 [3].

Ever since the description of Hinman syndrome in 1971, it has been entirely attributed to psychological and behavioral abnormalities. Abnormal family dynamics have been described as an association in about 50% of the cases [4]. However, a case series done in 2019 by Tiryaki et al. evaluating 12 children who fulfilled the criteria for Hinman syndrome with a 3 Tesla magnetic resonance imaging system and tractography of the lumbosacral plexus revealed abnormal findings resembling nerve injury series [5].

Hinman syndrome primarily presents with urinary incontinence and urinary tract infection. It typically peaks in late childhood and frequently resolves after puberty. Renal insufficiency has been associated with Hinman syndrome secondary to vesicoureteral reflux, obstruction, and infection [5]. 

The clinical criteria for the diagnosis of Hinman syndrome include intact perineal sensation and anal tone, normal lower extremity anatomy and function, absence of sacral skin lesions, normal radiographic evaluation of the lumbosacral spine, and lack of an abnormal MRI signal on MRI, CT, or myelography [6]. Urodynamic criteria supportive of Hinman syndrome include the inability to suppress bladder contractions, increased bladder capacity, and lack of evidence of detrusor denervation with normal detrusor reflex [6]. Radiologic findings include bladder trabeculations, or what is referred to as a “Christmas tree" bladder, vesicoureteral reflux, and an abnormal intravenous pyelogram (IVP). Dilatation of the posterior urethra has been described in approximately 50% of cases [7].

Since most children with Hinman syndrome are toilet trained initially and develop the aforementioned symptoms afterward, treatment should be multifactorial and should mainly focus on the behavioral aspects of the syndrome. Suggested treatment options include hypnosis as described by Hinman and Baumann, psychotherapy, retraining, bladder drill, biofeedback, and medications to prevent uninhibited bladder contractions including anticholinergic medications, diazepam, and phenoxybenzamine. Psychotropic medications have also been used in the treatment of Hinman syndrome. In addition, the treatment of fecal retention and superimposed urinary tract infections is crucial [3,4].

## Conclusions

In conclusion, although rare, Hinman syndrome should be considered in children presenting with symptoms of neurogenic bladder with associated behavioral abnormalities and without any underlying neurologic pathology. Multifactorial treatment addressing the psychological abnormalities as well as the urinary tract infections and fecal impaction can help prevent long-term renal damage. In addition, with the current advancements in imaging technologies, further study of subtle neurologic evaluation should be carried out in children with Hinman syndrome to deepen the understanding of this rare, but possibly devastating syndrome.

## References

[1] Manzo-Pérez G. (2014). Nonneurogenic neurogenic bladder (Hinman syndrome): Two different treatments for the same problem. Rev Mex Urol.

[2] Bauer SB (2017). The Hinman Syndrome. J Urol.

[3] Hinman F Jr (1986). Nonneurogenic neurogenic bladder (the Hinman syndrome)--15 years later. J Urol.

[4] Johnson JF 3rd, Hedden RJ, Piccolello ML, Wacksman J (1992). Distention of the posterior urethra: association with nonneurogenic neurogenic bladder (Hinman syndrome). Radiology.

[5] Tiryaki S, Eraslan C, Soyer T, Calli C, Ulman I, Avanoglu A (2019). Nonneuropathic neuropathic bladder-is it really nonneuropathic?. J Urol.

[6] Hinman F, Baumann FW (2017). Vesical and ureteral damage from voiding dysfunction in boys without neurologic or obstructive disease. J Urol.

[7] Phillips E, Uehling DT (1993). Hinman syndrome: a vicious cycle. Urology.

